# Education and prevalence of overweight and obesity among reproductive age group women in Ethiopia: analysis of the 2016 Ethiopian demographic and health survey data

**DOI:** 10.1186/s12889-020-08941-w

**Published:** 2020-07-31

**Authors:** Ayelign Mengesha Kassie, Biruk Beletew Abate, Mesfin Wudu Kassaw

**Affiliations:** College of Health Sciences, Department of Nursing, Woldia University, Woldia, Ethiopia

**Keywords:** Education, Ethiopia, Obesity, Overweight, Reproductive age group women

## Abstract

**Background:**

Globally, the prevalence of overweight and obesity is escalating, particularly among women and wealthier people. In many developed countries, overweight and obesity are more prevalent in persons with lower socioeconomic status. In contrast, studies in developing countries have reported a higher prevalence rate of overweight and obesity among women with higher educational status. Hence, this study aimed to assess the association between education and the prevalence of overweight and obesity among reproductive age group women in Ethiopia.

**Methods:**

This cross-sectional study was done based on the 2016 Ethiopian demographic and health survey (EDHS) data. From the total 15,683 women participants of the 2016 EDHS, 2848 reproductive age group women aged 15–49 years old who had a complete response to all variables of interest were selected and retained for analysis. Data were analyzed using SPSS version 20 software program. Both descriptive and logistic regression models were used for analysis.

**Results:**

The prevalence of overweight and obesity among the study participants was 11.5 and 3.4% respectively. The combined prevalence of overweight and obesity was 14.9%. From the total participants who are overweight and, or obese, majority, 83.3% were urban dwellers and the remaining 16.7% were rural dwellers. Education was positively associated with overweight and obesity among women. Besides, increased age, region, living in urban areas, being in rich quintile, increased frequency of watching television, and frequency of using internet were significantly associated with the odds of being overweight and obese among reproductive age group women in Ethiopia.

**Conclusions:**

The prevalence of overweight and obesity among reproductive age group women in Ethiopia is increasing compared to previous studies. Education was found to be a risk factor for overweight and obesity among women. Hence, context based interventions on the prevention and control methods of overweight and obesity are required.

## Background

Over nutrition is becoming the major global public health problem. It includes, overweight, obesity and diet-related non-communicable diseases (NCDs) [1]. Overweight and obesity refers an excessive fat accumulation in body tissues [2]. Obesity is an illness and necessitates immediate reversal to prevent early and untimely death among patients [2, 3].

Globally, the prevalence of overweight and obesity is escalating, particularly among women and wealthier people [4]. Overweight and obesity in women increases the risk of diabetes, hypertension, caesarean delivery, postpartum hemorrhage, and high birth weight baby, infant overweight and obesity [1, 5].

The disease burden related to high BMI has increased starting from 1990. Since 1980, the prevalence of obesity has doubled in more than 70 countries and has continuously increased in most other countries. In 2015 alone, more than 603 million adults were obese worldwide [6].

In 2013, the prevalence of overweight and obesity among women was 37% worldwide which is slightly higher than the men (36%) [7]. According to a study on trends in obesity among adults in the United States, the prevalence of obesity alone in 2013–2014 was 40.4% among women which is significantly higher than the men’s prevalence rate (35%) [8].

Even though largely preventable, overweight and obesity are liked to more deaths than underweight [9]. In 2015, high BMI had caused an estimated 4 million deaths globally, and nearly 40% of these deaths occurred in persons who were not obese but high BMI. More than two thirds of deaths related to high BMI were due to cardiovascular diseases [6, 10].

Latest WHO reports also show that overweight and obesity are becoming the leading causes of death worldwide [1, 9]. Overweight and obesity affects all age groups of people both in developed and developing countries regardless of their socioeconomic status [1, 11].

According to a study on the global trends of overweight and obesity, 26.9% of adults in Africa are overweight and obese. It has also revealed that obesity was twice more common among women than men [12].

Over nutrition costs the world billions of dollars a year in lost opportunities for economic growth and lost investments in human capital associated with increased preventable morbidity and mortality rates in both children and adults [1, 13].

Ethiopia is not different. According to the 2016 EDHS report 22% of reproductive age group women were underweight, an 8% drop from 30% in the 2000 EDHS. However, the proportion of overweight and obesity among women has increased from 3% in 2000 to 8% in 2016 [14].

Overweight and obesity are associated with many factors including excessive consumption of alcohol, cigarette smoking and sedentary life style habits [15, 16]. Overweight and obesity are also linked with lower socioeconomic status [17, 18].

In many developed countries, women with a low level of educational status are two to three times more likely to be overweight and obese than those with a higher level of education [19]. This might be due to the failure of women to recognize the risks and the consequences of being overweight and obese as fatness is considered a symbol of beauty and prosperity in many societies [20, 21].

Therefore, education is the major tool to promote healthy behaviors and solve these problems [22, 23]. However, there are contrasting studies in developing countries which have reported a higher prevalence rate of overweight and obesity among women with higher educational status than their counter parts [24, 25]. Hence, this study aimed to assess the association between educational status and the prevalence of overweight and obesity among reproductive age group women in Ethiopia.

## Methods

### Study design and population

This cross-sectional study was done based on the 2016 EDHS data. The survey included a nationally representative sample of women (aged 15–49 years) and men (aged 15–59 years) from the nine regions and two administrative cities of the country [14]. However, the current study involved non-pregnant reproductive age group women only because, unlike men, overweight and obesity in women are more prevalent and are associated with multiple problems including increased risk of diabetes, hypertension, caesarean delivery, postpartum hemorrhage, high birth weight babies, and infant overweight and obesity [1]. Pregnant women were excluded, because, pregnancy nullifies the values of BMI and data about BMI was not collected among pregnant, and those women who have had a birth in the 2 months before the survey in the 2016 EDHS [14].

### Sampling technique

In the 2016 EDHS, a two stage stratified sampling technique was employed to select representative samples for the country as whole. The regions in the country were stratified into urban and rural areas. Then, samples of enumeration areas (EAs) were selected in each stratum in two stages. In the first stage, 645 EAs were selected with probability proportional to the EA size. The EA size is the number of residential households in the EA as determined in the 2007 Ethiopian Population and Housing Census. A household listing operation was implemented in the selected EAs, and the resulting lists of households served as the sampling frame for the selection of households in the second stage. In the second stage, a fixed number of 28 households per cluster were selected with an equal probability systematic selection from the newly created household listing. All women aged 15–49 years who were usual members of the selected households or who spent the night before the survey in the selected households were eligible for the female survey [14]. For the purpose of this study, from the total, 15,683 women participants of the 2016 EDHS, a sub-sample of 2848 reproductive age group women aged 15–49 years who had a complete response to all variables of interest were selected and retained for analysis after excluding women who were pregnant.

### Data collection

In the 2016 EDHS, a standardized and validated questionnaire were adapted from the DHS Program’s standard Demographic and Health Survey questionnaires in a way to reflect the population and health issues relevant to Ethiopia. The survey data were collected from January 18 to June 27, 2016 by trained field workers [14]. For the purpose of the current study, the women’s data from the 2016 EDHS was utilized.

### Variables and operational definitions

**Fig. 1:**
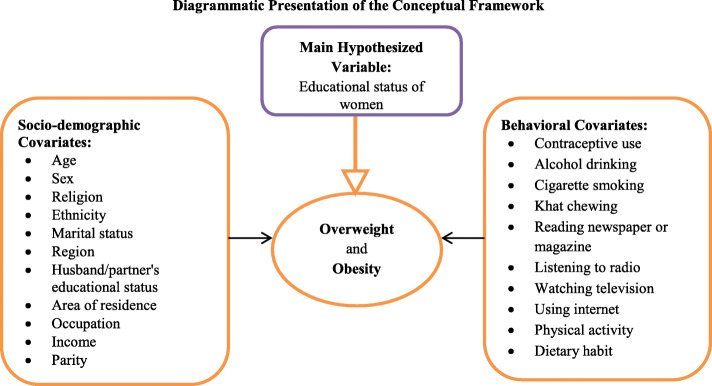
Conceptual framework for the prevalence of overweight and obesity and its associated factors among reproductive age group women in Ethiopia, adapted from different sources by the principal investigator after reviewing different literatures [8, 14, 26, 27]

In addition to education, several important covariates like, respondent’s age, and wealth index were analyzed depending on their availability in the 2016 EDHS data (Fig 1). ***Educational*** level of participants was categorized as; no education, primary, secondary, and higher education. Additional details of independent variables are available somewhere else [28]. The dependent variables of interest were overweight and obesity among non-pregnant women aged 15–49 years. These outcome variables of interest were categorized as follows based on the WHO Classification of body mass index; overweight if the BMI is 25.0–29.9 kg/m^2^ and obese if it is≥30 kg/m^2^ [2]. The combined prevalence of overweight and obesity was determined by merging the two outcomes together.

### Statistical analysis

Data analysis started with a summary of the socio-demographic characteristics, and other important factors were included in assessment of overweight and obesity among women using frequency distribution analysis. Weighting was applied during preparation of analytical sample and in all percentage calculations so that to make the results representative for reproductive age group women in Ethiopia. Data were analyzed using SPSS version 20 software program. Bivariate analysis using Pearson’s chi-squared test was used to assess the frequency distribution of the main outcomes and is presented in relation to different socio-demographic characteristics. Binary logistic regression analysis was carried out to see the association between overweight and obesity and each predictor variable separately to present a crude or unadjusted analysis. Then, a multivariable logistic regression analysis was done to examine the association between educational status of women, other covariates and overweight and, or obesity. A *p-value* of less than 0.05 was used to declare a statistically significant association between the independent and the outcome variables.

## Result

### Baseline characteristics of respondents

In this study 2848 participants of the 2016 EDHS were included. More than half, 53.2% of the participants were Orthodox Christianity followers, 23.7% Muslims, 21.9% Protestants, 0.7% Catholics and the remaining 0.5% were traditional and other religion followers. Regarding to educational status of the study participants, 43.2% had no education and the remaining 56.8% had completed up to higher levels of education. Majority, 63% of the participants were rural dwellers. Furthermore, 59.6% of the participants were in the first (rich) wealth index quintile, 15.9% were in the second (middle) quintile and 24.5% were in the third (poor) quintile (Table 1).

**Table 1:** Sociodemographic characteristics of reproductive age group women in Ethiopia, EDHS 2016, (*N* = 2848)

Characteristics	Response	Frequency	Percentage
Educational status of respondents	No education	1231	43.2
	Primary	990	34.8
	Secondary	365	12.8
	Higher	262	9.2
Respondents age	15–24 years old	728	25.6
	25-34 years old	1306	45.9
	> = 35 years old	814	28.6
Religion	Orthodox	1516	53.2
	Catholic	21	0.7
	Protestant	623	21.9
	Muslim	674	23.7
	Others	14	0.5
Region of respondents	Tigray	330	11.6
	Afar	70	2.5
	Amhara	532	18.7
	Oromia	363	12.7
	Somali	16	0.6
	Benishangul	207	7.3
	SNNPR	480	16.9
	Gambella	192	6.7
	Harari	146	5.1
	Addis Ababa	354	12.4
	Dire Dawa	158	5.5
Area of residence	Urban	1054	37.0
	Rural	1794	63.0
Employment status	Not employed	1243	43.6
	Employed	1605	56.4
Husband/partners educational status	No education	899	31.6
	Primary	1089	38.2
	Secondary	465	16.3
	Higher	395	13.9
Respondents wealth index	Poor	698	24.5
	Medium	453	15.9
	Rich	1697	59.6

The prevalence of overweight and obesity among reproductive age group women was 11.5 and 3.4% respectively and, the combined prevalence of overweight and obesity was 14.9% (Table 2).

**Table 2:** Prevalence of overweight and obesity among reproductive age group women in Ethiopia (*N* = 2848)

Variable	Outcome	Frequency	Percentage
Overweight	Yes	327	11.5
	No	2521	88.5
Obese	Yes	98	3.4
	No	2750	96.6
Overweight and obese	Yes	425	14.9
	No	2423	85.1

The prevalence of overweight and obesity was higher, 37, 26.7 and 16.2% respectively among women with a higher, secondary and primary level of education compared to 5.8% among women with no education. Besides, the prevalence of overweight and obesity was 19.8 and 16.2% among women with age group of > = 35 years and 25–34 years respectively compared to 6.6% in women with age group of 15–24 years. Similarly, the prevalence of overweight and obesity was also higher, 23.7% in women with rich wealth quintile compared to 1.4% in women with poor wealth quintile (Table 3).

**Table 3:** Cross-tabulation of baseline characteristics, and overweight and obesity among reproductive age group women in Ethiopia (*N* = 2848)

Respondents baseline Characteristics	Overweight and Obese		*P*-Value
	Yes, n (%)	No, n (%)	
Educational status of respondents			
No education	72(5.8)	1159(94.2)	< 0.001
Primary	160(16.2)	830(83.8)	
Secondary	96(26.7)	269(73.3)	
Higher	97(37.0)	165(63.0)	
Respondents age			
15–24 years old	48(6.6)	680(93.4)	< 0.001
25-34 years old	216(16.5)	1090(83.5)	
> =35 years old	161(19.8)	653(80.2)	
Religion			
Orthodox	254(16.8)	1262(83.2)	< 0.001
Catholic	2(9.5)	19(90.5)	
Protestant	55(8.8)	568(91.2)	
Muslim	113(16.8)	561(83.2)	
Others	1(7.1)	13(92.9)	
Number of children			
None	24(10)	215(90)	< 0.001
1–3	299(18.3)	1335(81.7)	
> =4	102(10.5)	873(89.5)	
Type of current contraceptive use			
Traditional	23(31.5)	50(68.5)	< 0.001
Modern	402(14.5)	2373(85.5)	
Duration of current contraceptive use			
< =6 month	258(15.5)	1408(84.5)	0.316
> 6 month	167(14.1)	1015(85.9)	
Region of respondents			
Tigray	22(6.7)	308(93.3)	< 0.001
Afar	16(22.9)	54(77.1)	
Amhara	23(4.3)	509(95.7)	
Oromia	33(9.1)	330(90.9)	
Somali	5(31.2)	11(68.8)	
Benishangul	26(12.6)	181(87.4)	
SNNPR	30(6.2)	450(93.8)	
Gambella	22(11.5)	170(88.5)	
Harari	51(34.9)	95(65.1)	
Addis Ababa	147(41.5)	207(58.5)	
Dire Dawa	50(31.6)	108(68.4)	
Residence			
Urban	354(33.6)	700(66.4)	< 0.001
Rural	71(4.0)	1723(96.0)	
Employment status			0.223
Not employed	174(14.0)	1069(86.0)	
Employed	251(15.6%)	1354(84.4)	
Husband/partners educational status			
No education	57(6.3)	842(93.7)	< 0.001
Primary	117(10.7)	972(89.3)	
Secondary	124(26.7)	341(73.3)	
Higher	127(32.2)	268(67.8)	
Respondents wealth index			< 0.001
Poor	10(1.4)	688(98.6)	
Medium	13(2.9)	440(97.1)	
Rich	402(23.7)	1295(76.3)	
Respondent drinks alcohol			
No	228(14.3)	1364(85.7)	0.311
Yes	197(15.7)	1059(84.3)	
Respondent smokes cigarette			
No	423(14.9)	2408(85.1)	0.714
Yes	2(11.8)	15(88.2)	
Respondent chews chat			
No	347(13.6)	2199(86.4)	< 0.001
Yes	78(25.8)	224(74.2)	
Frequency of reading newspaper or magazine			
Not at all	267(11.3)	2095(88.7)	< 0.001
Less than once a week	111(30.9)	248(69.1)	
At least once a week	47(37.0)	80(63.0)	
Frequency of listening to radio			
Not at all	185(10.9)	1514(89.1)	< 0.001
Less than once a week	95(17.1)	459(82.9)	
At least once a week	145(24.4)	450(75.6)	
Frequency of watching television			
Not at all	85(5.1)	1572(94.9)	< 0.001
Less than once a week	53(14.0)	325(86.0)	
At least once a week	287(35.3)	526(64.7)	
Frequency of using internet			
Not at all	351(13.1)	2326(86.9)	< 0.001
Less than once a week	16(42.1)	22(57.9)	
At least once a week	32(44.4)	40(55.6)	
Almost every day	26(42.6)	35(54.7)	

### Regression analysis of associated factors with overweight and obesity

In this study bivariate logistic regression analysis was performed and, variables which have a *p*-value of less than 0.25 were fitted into the multivariable logistic regression analysis model. In the multivariable logistic regression analysis, educational status was significantly associated the odds of being overweight and obese among women. The odds of being overweight and obese was around 2 times higher among those who had higher (AOR = 2.11, 95% CI, 1.18, 3.76), secondary (AOR = 1.90, 95% CI, 1.16, 3.12) and primary education (AOR = 1.91, 95% CI, 1.30, 2.79) than those who had no education.

Besides, respondent’s age, region, residence, wealth index, frequency of watching television, and frequency of using internet were positively associated with the odds of overweight and obesity among women. The odds of overweight and obesity among respondents aged 25–34 years and > =35 years was more than 3 (AOR = 3.03, 95% CI, 2.06, 4.48) and 6.5 (AOR = 6.51, 95% CI, 4.08, 10.37) times higher respectively than those aged 15–24 years old. The odds of being overweight and obese among respondents living in Oromia, Somali, Benishangul, Harari, Addis Ababa, and Dire Dawa was higher than those who live in Tigray region.

Similarly, the odds of those who live in urban areas was more than 3 folds higher (AOR = 3.11, 95% CI, 2.02, 4.80) than those who live in rural areas. Wealth index was also significantly associated with overweight and obesity. The odds of being overweight and obese among respondents of rich wealth quintile class was around 5 times higher (AOR = 4.82, 95% CI, 2.40, 9.71) than those who are from poor wealth quintile class. Frequency of media use was also significantly associated with overweight and obesity. The odds of being overweight and obese was higher among those watch television at least once a week (AOR = 1.91, 95% CI, 1.26, 2.90) and use internet at least once a week (AOR = 1.89, 95% CI, 1.06, 3.36) compared to those who did not use it at all (Table 4).

**Table 4:** Regression analysis of overweight and obesity among reproductive age group women with their educational status and other covariates in Ethiopia, (*N* = 2848)

Respondent Characteristics	Overweight and Obese		Bivariate logistic regression	Multivariable logistic regression	*P*-Value
	Yes, n (%)	No, n (%)	COR (95%CI)	AOR (95%CI)	
Educational status					
No education	72(5.8)	1159(94.2)	1	1	
Primary	160(16.2)	830(83.8)	3.10(2.32,4.16)	**1.91(1.30,2.79)**	0.001
Secondary	96(26.7)	269(73.3)	5.75(4.12,8.02)	**1.90(1.16,3.12)**	0.011
Higher	97(37.0)	165(63.0)	9.46(6.70,13.37)	**2.11(1.18,3.76)**	0.012
Respondents age					
15–24 years old	48(6.6)	680(93.4)	1	1	
25-34 years old	216(16.5)	1090(83.5)	2.81(2.02,3.89)	**3.03(2.06,4.48)**	< 0.001
> =35 years old	161(19.8)	653(80.2)	3.49(2.49,4.91)	**6.51(4.08,10.37)**	< 0.001
Religion					
Orthodox	254(16.8)	1262(83.2)	1	1	
Catholic	2(9.5)	19(90.5)	0.52(0.12,2.26)	0.46(0.09,2.37)	0.353
Protestant	55(8.8)	568(91.2)	0.48(0.35,0.66)	0.72(0.48,1.07)	0.105
Muslim	113(16.8)	561(83.2)	1.00(0.79,1.28)	1.01(0.73, 1.39)	0.977
Others	1(7.1)	13(92.9)	0.38(0.05,2.94)	0.87(0.10,7.69)	0.901
Number of children					
None	24(10)	215(90)	1	1	
1–3	299(18.3)	1335(81.7)	2.01(1.29,3.12)	1.46(0.86,2.49)	0.167
> =4	102(10.5)	873(89.5)	1.05(0.66,1.67)	1.496(0.78,2.89)	0.229
Type of current contraceptive use					
Traditional	23(31.5)	50(68.5)	2.72(1.64,4.50)	0.74(0.40,1.36)	0.335
Modern	402(14.5)	2373(85.5)	1	1	
Respondents region					
Tigray	22(6.7)	308(93.3)	1	1	
Afar	16(22.9)	54(77.1)	4.15(2.05,8.40)	2.06(0.90,4.71)	0.087
Amhara	23(4.3)	509(95.7)	0.63(0.35,1.15)	0.96(0.49,1.86)	0.896
Oromia	33(9.1)	330(90.9)	1.40(0.80,2.45)	**2.29(1.21,4.35)**	0.011
Somali	5(31.2)	11(68.8)	6.36(2.03,19.94)	**4.88(1.30,18.31)**	0.019
Benishangul	26(12.6)	181(87.4)	2.01(1.11,3.65)	**4.05(2.04,8.07)**	< 0.001
SNNPR	30(6.2)	450(93.8)	0.93(0.53,1.65)	1.89(0.96,3.74)	0.067
Gambella	22(11.5)	170(88.5)	1.81(0.98,3.37)	1.54(0.77,3.09)	0.227
Harari	51(34.9)	95(65.1)	7.52(4.34,13.03)	**3.10(1.62,5.92)**	0.001
Addis Ababa	147(41.5)	207(58.5)	9.94(6.14,16.09)	**2.54(1.46,4.39)**	0.001
Dire Dawa	50(31.6)	108(68.4)	6.48(3.75,11.20)	**2.28(1.21,4.29)**	0.011
Residence					
Urban	354(33.6)	700(66.4)	12.27(9.37,16.07)	**3.11(2.02,4.80)**	< 0.001
Rural	71(4.0)	1723(96.0)	1	1	
Employment status					
Not employed	174(14.0)	1069(86.0)	0.88(0.71,1.08)	0.89(0.68,1.15)	0.361
Employed	251(15.6%)	1354(84.4)	1	1	
Husband/partners educational status					
No education	57(6.3)	842(93.7)	1	1	
Primary	117(10.7)	972(89.3)	1.78(1.28,2.47)	0.83(0.56,1.24)	0.365
Secondary	124(26.7)	341(73.3)	5.37(3.83,7.53)	1.00(0.63,1.59)	0.999
Higher	127(32.2)	268(67.8)	7.00(4.98,9.85)	1.11(0.67,1.84)	0.697
Wealth index					
Poor	10(1.4)	688(98.6)	1	1	
Medium	13(2.9)	440(97.1)	2.03(0.88,4.68)	1.99(0.85,4.63)	0.111
Rich	402(23.7)	1295(76.3)	21.36(11.33,40.27)	**4.82(2.40,9.71)**	< 0.001
Respondent chews Chat					
No	423(14.9)	2408(85.1)	1	1	
Yes	2(11.8)	15(88.2)	2.21(1.67,2.92)	1.42(0.98,2.07)	0.063
Frequency of reading newspaper or magazine					
Not at all	267(11.3)	2095(88.7)	1	1	
Less than once a week	111(30.9)	248(69.1)	3.51(2.72,4.54)	1.03(0.73,1.46)	0.853
At least once a week	47(37.0)	80(63.0)	4.61(3.15,6.76)	1.08(.67,1.75)	0.760
Frequency of listening to radio					
Not at all	185(10.9)	1514(89.1)	1	1	
Less than once a week	95(17.1)	459(82.9)	1.69(1.30,2.22)	**0.63(0.45,0.89)**	0.008
At least once a week	145(24.4)	450(75.6)	2.64(2.07,3.36)	0.85(0.61,1.17)	0.320
Frequency of watching television					
Not at all	85(5.1)	1572(94.9)	1	1	
Less than once a week	53(14.0)	325(86.0)	3.02(2.10,4.34)	1.32(0.83,2.12)	0.246
At least once a week	287(35.3)	526(64.7)	10.09(7.77,13.11)	**1.91(1.26,2.90)**	0.002
Frequency of using internet					
Not at all	351(13.1)	2326(86.9)	1	1	
Less than once a week	16(42.1)	22(57.9)	4.82(2.51,9.27)	1.33(0.62,2.86)	0.461
At least once a week	32(44.4)	40(55.6)	5.30(3.29,8.55)	**1.89(1.06,3.36)**	0.031
Almost every day	26(42.6)	35(54.7)	4.92(2.93,8.28)	1.28(0.68,2.43)	0.449

## Discussion

The prevalence of overweight and obesity among the study participants was 11.5 and 3.4% respectively. The combined prevalence of overweight and obesity was 14.9%. This finding is higher than previous studies conducted in Ethiopia [29, 30]. However, it is lower than a study in Malawi that the prevalence of overweight and obesity among adult women was 16.8 and 6.3%, respectively [26]. This variation in the prevalence rates might be due to the differences in the age group of study participants.

Unlike the current study, the Malawi study was conducted among adults aged from 18 years to 49 years old. The current study was conducted among reproductive age group women aged from 15 to 49 years old which can potentially lower the prevalence of overweight and obesity among women. Because, age of participants was one determinant which was significantly associated with the prevalence of overweight and obesity among the study participants. The prevalence of overweight and obesity among the age group of participants containing adolescents was lower than the other groups in this study. Adolescence is a stage of rapid growth and development and there is an increase in nutrition demand at this time [31]. Thus, the inclusion of adolescents may potentially hider the overall prevalence of overweight and obesity among the study participants in this study.

It is also lower than a study in India which had shown that the prevalence of overweight and obesity among reproductive age group women was 22.6 and 10.7% respectively [27]. This variation may emanate from differences in developmental level of the two countries, because, majority of the associated factors with overweight and obesity are the result of demogtaphic and socioeconomic transitions across countries [32]. The high prevalence of overweight and, or obesity in women might be also due to their physiology as they tend to deposit more fat than lean mass [33–35].

Besides, from the total participants who are overweight and obese, majority, 83.3% were urban dwellers and the remaining 16.7% were rural dwellers. This finding is in line with the Malawi and Indian studies that the prevalence of overweight and obesity was higher among women living in urban areas as compared to their counter parts [26, 27]. Consistent finding has been also reported in other low and middle-income countries [36–38]. This could be attributed to the life style of urban dwellers. Unlike rural residents who are usually more actively involved in a less sedentary lifestyle and more laborious activities, the occupation of urban dwellers may result in sedentary type life styles among women [39–41].

In the multivariable logistic regression analysis model, educational status of women was positively associated with the odds of being overweight and obese. The odds of being overweight and obese was around 2 times higher among those who had higher, secondary and primary educational status than those who had no education. This is not expected because people who are educated are expected to get more information on the effect and methods of overweight and obesity from different sources than those who are not educated. Nevertheless, consistent findings have been reported in other low and middle-income countries [24, 25]. The possible explanation could be people with a higher level of educational status usually live in urban areas where the prevalence of overweight and obesity is higher than those who are not educated [42].

Besides, women with higher educational level are more likely to have a higher wealth status one of the significant factor for overweight and obesity than less-educated women [43]. The other reason could be as people with better educational status usually live in urban areas, the nature of their occupation might lead to increased body weight than the rural residents [42, 44]. This can be justified by the results of the current study that from the total participants who are overweight and obese, majority, 83.3% were urban dwellers and only 16.7% were rural dwellers.

In addition, respondent’s age, region, residence, wealth index, frequency of listening to radio, frequency of watching television, and frequency of using internet were significantly associated with overweight and obesity among women. The odds of being overweight and obese among respondents aged 25–34 years old and > =35 years old were more than 3 and 6.5 times higher respectively than those aged 15–24 years old. This is in agreement with studies conducted in both developed and developing countries which have indicated that overweight and obesity in women tend to increase with age [45–47].

It might be also due to family transitions as some studies shown that starting at the age of 30 years women tend to shift various household roles to their children making themselves less active in routine household activities. As women grew old, some tend to express less willingness to reduce their body weight irrespective of their health status [47]. The link between age and body weight might be due to the fact that increasing age is a known risk factor of overweight, obesity and other non-communicable diseases [48]. Furthermore, advancing age is linked with number of parity which is an important risk factor for overweight and obesity [49]. Body weight usually increases during pregnancy which can be continued for a lifetime if weight loss did not occur in the post-partum period [50, 51].

The odds of being overweight and obese among respondents living in Oromia, Somali, Benishangul, Harari, Addis Ababa, and Dire Dawa was higher than those who live in Tigray region. This difference might be due to differences in sociodemographic and socioeconomic status of the people in these regions. For example, unlike Tigray region which contains both urban and rural residents, Addis Ababa and Dire Dawa are urban and people living in urban areas are at increased risk of being overweight and obese as evidenced in this and other previous studies [26, 27, 52, 53].

In this study the odds of being overweight and obese was more than 3 folds higher among women living in urban areas than those who live in rural areas. This finding is in line with the Malawi and Indian studies [26, 27]. This could be due to the fact that urban dwellers are usually from middle and high income groups of people and, households with a high income tend to purchase food in bulk, spending more on both healthy and less healthy foods [47]. The other reason could be due to differences in the life style of urban dwellers. Unlike the rural residents who are usually more actively involved in a less sedentary lifestyle and more laborious activities, the occupation of urban dwellers may result in sedentary type life styles [39].

Similarly, in this study, wealth index was significantly associated with increased odds of overweight and obesity. The odds of being overweight and obese among respondents of rich wealth quintile class was around 5 times higher than those who are from poor wealth quintile class. This finding is consistent with a study in Kenya where the women with more sedentary life style were in the highest income groups [54]. It is also consistent with a study in Bangladesh [43]. This might be also due to their capacity of purchasing more energy-dense foods as witnessed from studies in both developed and developing countries that high-income households purchased foods in bulk and were more likely to over consume these foods [13, 55].

Besides, frequency of media use was also significantly associated with overweight and obesity. The odds of being overweight and obese was a round 2 times higher among those who watch television at least once a week and use internet at least once a week respectively compared to those who did not use it at all. The possible explanation is that people who use media are more likely from medium and rich groups a very significant factor which is associated with overweight and obesity in this and other studies [26, 27]. In addition, education was also associated with the odds of overweight and obesity. The other reason is that people who use media are usually urban dwellers, another highly significant determinant of overweight and obesity [26, 27, 44].

However, in this study smoking, alcohol consumption, chewing chat and contraceptive use were not associated with the odds of being overweight and obese. This finding is inconsistent compared with a study in India which found that use of hormonal contraceptives was one of the significant factors that determine the prevalence of overweight and obesity among women [27]. This is unexpected, because, hormonal contraceptives are a known risk factor for overweight and obesity and requires further studies to affirm the association between contraceptive use and overweight and obesity among women in Ethiopia [56–58].

### Strength and limitations of the study

One of the main strength of this study is its large sample size and the random sampling method used to recruit participants in the survey which makes the study participants a true representative of reproductive age group non-pregnant women in Ethiopia. The quality of the data is also assured as the EDHS uses well-trained field personnel’s, a standardized protocol, and validated tools in the data collection process. However, as a limitation, some of the very important determinants of overweight and obesity such as physical activity and dietary habits were not included in this study because, the relevant information’s regarding these variables are not available in the 2016 EDHS dataset. The other important limitation is the information in the survey was self-reported, so some degree of under-reporting of socially unacceptable behaviors and over-reporting of socially desirable behaviors are likely [14].

## Conclusion

The prevalence of overweight and obesity among reproductive age group women is increasing in Ethiopia particularly among urban dwellers. Education was positively associated with the odds of overweight and obese among women. Besides, increased age, region, residing in urban areas, being rich, increased frequency of watching television, and frequency of using internet were significantly associated with overweight and obesity. This is an indication of how overweight and obesity are major problems for women especially to those living in urban areas regardless of their educational status. This is worrying because overweight and obesity might increase women’s vulnerability to different type of non-communicable diseases including hypertension and other cardiovascular diseases. Thus, context based interventions need to be designed on the prevention and control methods of overweight and obesity giving especial emphasis to those residing in urban areas.

## Data Availability

Data supporting the conclusions of this article are included within the article and its supporting files.

## Abbreviations

BMI: Body Mass Index
CI: Confidence interval
DHS: Demographic and Health Surveys
EDHS: Ethiopian Demographic and Health Survey
ICF: Inner City Fund
OR: Odds Ratio
SD: Standard deviation
SNNPR: Southern Nations, Nationalities, and Peoples’ Region
WHO: World Health Organization
